# Physical Activity as a Vital Sign: A Systematic Review

**DOI:** 10.5888/pcd14.170030

**Published:** 2017-11-30

**Authors:** Yvonne M. Golightly, Kelli D. Allen, Kirsten R. Ambrose, Jamie L. Stiller, Kelly R. Evenson, Christiane Voisin, Jennifer M. Hootman, Leigh F. Callahan

**Affiliations:** 1 Thurston Arthritis Research Center, University of North Carolina at Chapel Hill, Chapel Hill, North Carolina; 2 Department of Epidemiology, Gillings School of Global Public Health, University of North Carolina at Chapel Hill, Chapel Hill, North Carolina; 3 Injury Prevention Research Center, University of North Carolina at Chapel Hill, Chapel Hill, North Carolina; 4 Health Services Research & Development, VA Medical Center, Durham, North Carolina; 5 Department of Medicine, University of North Carolina at Chapel Hill, Chapel Hill, North Carolina; 6 The Cecil B. Sheps Center for Health Services Research, University of North Carolina at Chapel Hill, Chapel Hill, North Carolina; 7 Arthritis Program, Centers for Disease Control and Prevention, Atlanta, Georgia; 8 Departments of Social Medicine and Orthopaedics, University of North Carolina at Chapel Hill, Chapel Hill, North Carolina

## Abstract

**Introduction:**

Physical activity (PA) is strongly endorsed for managing chronic conditions, and a vital sign tool (indicator of general physical condition) could alert providers of inadequate PA to prompt counseling or referral. This systematic review examined the use, definitions, psychometric properties, and outcomes of brief PA instruments as vital sign measures, with attention primarily to studies focused on arthritis.

**Methods:**

Electronic databases were searched for English-language literature from 1985 through 2016 using the terms PA, exercise, vital sign, exercise referral scheme, and exercise counseling. Of the 838 articles identified for title and abstract review, 9 articles qualified for full text review and data extraction.

**Results:**

Five brief PA measures were identified: Exercise Vital Sign (EVS), Physical Activity Vital Sign (PAVS), Speedy Nutrition and Physical Activity Assessment (SNAP), General Practice Physical Activity Questionnaire (GPPAQ), and Stanford Brief Activity Survey (SBAS). Studies focusing on arthritis were not found. Over 1.5 years of using EVS in a large hospital system, improvements occurred in relative weight loss among overweight patients and reduction in glycosylated hemoglobin among diabetic patients. On PAVS, moderate physical activity of 5 or more days per week versus fewer than 5 days per week was associated with a lower body mass index (−2.90 kg/m^2^). Compared with accelerometer-defined physical activity, EVS was weakly correlated (*r *= 0.27), had low sensitivity (27%–59%), and high specificity (74%–89%); SNAP showed weak agreement (κ = 0.12); GPPAQ had moderate sensitivity (46%) and specificity (50%), and SBAS was weakly correlated (*r* = 0.10–0.28), had poor to moderate sensitivity (18%–67%), and had moderate specificity (58%–79%).

**Conclusion:**

Few studies have examined a brief physical activity tool as a vital sign measure. Initial investigations suggest the promise of these simple and quick assessment tools, and research is needed to test the effects of their use on chronic disease outcomes.

## Introduction

Arthritis is a common cause of disability in the United States, affecting at least 52 million Americans (1,2). Physical activity (PA) improves pain and physical function for people with arthritis (3–5), and a lack of PA is linked with the premature development of chronic diseases (6). The importance of PA for better health and weight management is a well-known public health message (7), yet less than 10% of adults in the United States achieve the 2008 US Department of Health and Human Services PA guidelines (≥150 min/wk of moderate PA or ≥75 min/wk of vigorous PA, or equivalent combination of the 2) (8), and most people with arthritis do not achieve these recommended PA levels (9,10).

Recent efforts (eg, American College of Sports Medicine’s Exercise is Medicine, Surgeon General’s Step It Up!) encourage the medical community to promote PA for disease prevention and management (11). These programs urge primary care physicians and other health care professionals to assess PA during patient encounters and prescribe exercise in treatment plans. To fulfill this initiative, the first line of action from the health care community is to ask patients about their PA habits. Data on PA can be obtained during clinical encounters as a vital sign (indicator of general physical condition). This vital sign alerts the provider of inadequate PA, prompting exercise counseling or referral to enhance chronic disease management. Few health systems use PA vital sign alerts, and no review has examined available PA vital sign tools.

The purpose of this study was to perform a systematic review of the definitions, use, psychometric properties, and outcomes of instruments examining PA as a vital sign in clinical settings. Studies related to arthritis were the focus; other chronic diseases were assessed. We discuss knowledge gaps and considerations that may advance the use of these instruments as a PA vital sign.

## Methods

Our literature search was conducted following the Preferred Reporting Items for Systematic Reviews and Meta-Analyses (PRISMA) guidelines (12). Using electronic sources of PubMed/Medline, Cochrane, CINAHL, and Global Health, a research librarian (C.V.) performed searches (1985–present) in March 2014 and July 2015 for English-language articles in scientific journals of human adults (aged ≥18 y) using the terms “physical activity,” “exercise,” “vital sign,” “exercise referral scheme,” and “exercise counseling.” Our initial search was limited to the term “arthritis,” but yielded no results, and the term was thus removed. Because few articles were eligible for inclusion from these searches (3 articles), and because of the rising interest in this area, we monitored the literature for new publications into 2016. In February and August 2016, searches using the same terms were conducted by one author (Y.M.G.) to locate articles not identified by prior searches.

Initial inclusion criteria were a focus on PA or exercise and evidence that PA was considered as a vital sign or intended for use in a clinical setting as a screening tool for inadequate PA. Articles were included if the research design was a randomized or quasi-randomized clinical trial, observational study, pooled data analysis, meta-analysis, or systematic review, or if psychometric properties of the instruments were tested. Case series reports, case reports, nonsystematic review articles, editorials, and letters to the editor were excluded.

Two authors (Y.M.G. and K.D.A.) performed independent review of the identified abstracts, and disagreements between reviewers were resolved by consensus. Next, these authors reviewed full-text articles and confirmed with another author (L.F.C.) which articles to include for data extraction. One author (Y.M.G.) reviewed bibliographies for all articles during full text review to identify additional relevant articles. Data extraction was conducted independently by reviewers (Y.M.G., J.L.S., or K.R.A.) using a spreadsheet with categories approved by all authors. These categories included basic article information (first author, publication year, title, study years, country), information related to use of a PA vital sign tool (system/environment, target population, provider type, parent study or data source, study design, intervention, number of participants, age distribution of participants, proportion female, proportion by race/ethnicity, body mass index [BMI] distribution of participants, arthritis or other comorbid chronic conditions), and definitions, psychometric properties, and outcomes of the tool (primary and secondary outcome measures). Because this review was descriptive and did not emphasize trials solely, we did not assess risk of bias or study quality or plan to combine data and statistical analyses.

## Results

### Literature search

Because the search terms were broad, the electronic search during March 2014 located 454 articles (Figure). On the basis of the title and abstract review, 3 articles were identified and were included in full-text review. The July 2015 electronic search located 338 new articles of which none met inclusion criteria. Of the 46 new articles identified by the February 2016 search, 5 articles met inclusion criteria and were accessible for full-text review. Of the 10 new articles identified by the August 2016 search, 1 article met inclusion criteria and was included for full-text review. No additional articles were identified from bibliographies. Authors agreed to include 9 articles using 5 vital sign tools during the data extraction phase (13–21). No articles focused on arthritis; only 1 included participants specifically with arthritis (18). 

**Figure:**
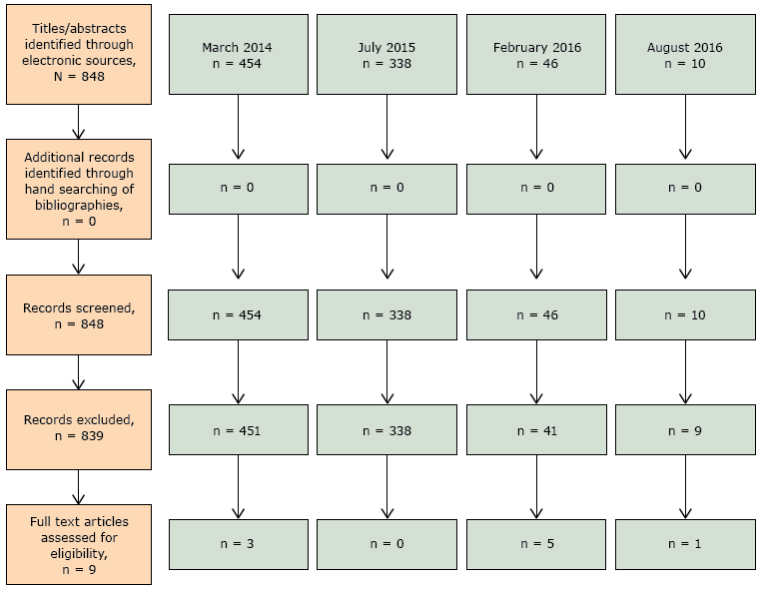
Flow diagram of article selection, review of physical activity instruments as vital signs, 1985–2016.

### Identified Brief Physical Activity Vital Sign Tools and definitions of physical activity

The Exercise Vital Sign (EVS) (16–18,20,21), the Physical Activity Vital Sign (PAVS) (13–15,19), the PA component of the Speedy Nutrition and Physical Activity Assessment (SNAP) (13), the General Practice Physical Activity Questionnaire (GPPAQ) (17), and the Stanford Brief Activity Survey (SBAS) (20) were the brief PA vital sign instruments identified during this review. All 5 tools (Table 1) assessed moderate to vigorous PA in adults.

**Table 1:** Summary of Physical Activity Measures, Review of Physical Activity Instruments as Vital Signs, 1985–2016

Physical Activity Measure	No. of Items	Time	Items	Responses	Scoring/Interpretation
Exercise Vital Sign (EVS)	2	<30 sec	1. On average, how many days per week do you engage in moderate to strenuous exercises (like a brisk walk)? 2. On average, how many minutes per day do you exercise at this level?	1. Record number of days (0–7) 2. 7 Categories: 10, 20, 30, 40, 50, 60, 90, 120, and ≥150 min	Multiply the responses to both questions to estimate number of minutes per week of moderate to strenuous exercise. This score is used to determine whether the patient achieves the recommended amount of 150 minutes of moderate to vigorous physical activity per week.
Physical Activity Vital Sign (PAVS)	2	<30 sec	1. How many days during the past week have you performed physical activity where your heart beats faster and your breathing is harder than normal for 30 minutes or more? 2. How many days in a typical week do you perform activity such as this?	1. Record number of days (0–7)	Score presented as days during the past week over days in typical week (range, 0/0 to 7/7).
Speedy Nutrition and Physical Activity Assessment (SNAP), physical activity component	1	<1 min	1. Are you active for 30 minutes on 5 days of the week? Examples of activity are: • walking • housework • work in the yard or garden • dancing • jobs that require walking, lifting or other hard work • exercise	Circle one number only: 1. No, and I have no plans to be more active. 2. No, but I have been thinking about being more active. 3. Sometimes I am active for 30 minutes, but not all the time. 4. Yes, I am active for 30 minutes on 5 days of the week.	A score of 4 (ie, chose item 4) is classified as sufficiently active.
General Practice Physical Activity Questionnaire (GPPAQ)	7	60 sec	1. Please tell us the type and amount of physical activity involved in your work. 2. During the last week, how many hours did you spend on each of the following activities? 3. How would you describe your usual walking pace?	1. Select one of the following: 1a. I am not in employment (eg, retired, retired for health reasons, unemployed, full-time carer). 1b. I spend most of my time at work sitting (such as in an office). 1c. I spend most of my time at work standing or walking. However, my work does not require much intense physical effort (eg, shop assistant, hairdresser, security guard, childminder). 1d. My work involves definite physical effort including handling of heavy objects and use of tools (eg, plumber, electrician, carpenter, cleaner, hospital nurse, gardener, postal delivery workers). 1e. My work involves vigorous physical activity including handling of very heavy objects (eg, scaffolder, construction worker, refuse collector). 2. For each item (2a–2e) select 1 of these 4 responses: none, some but less than 1 hour, 1 hour but less than 3 hours, 3 hours or more. 2a. Physical exercise such as swimming, jogging, aerobics, football, tennis, gym workout. 2b. Cycling, including cycling to work and during leisure time. 2c. Walking, including walking to work, shopping, for pleasure. 2d. Housework/childcare 2e. Gardening/do it yourself 3. Select one of the following: Slow pace (ie, less than 3 mph), steady average pace, brisk pace, fast pace (ie, over 4 mph).	Inactive = sedentary job and no physical exercise or cycling. Moderately inactive = sedentary job and some but <1 hour of physical exercise and/or cycling per week OR standing job and no physical exercise or cycling. Moderately active = sedentary job and 1.0–2.9 hours of physical exercise and/or cycling per week OR standing job and some but <1 hour of physical exercise and/or cycling per week OR physical job and no physical exercise or cycling. Active = sedentary job and ≥3 hours of physical exercise and/or cycling per week OR standing job and 1.0–2.9 hours of physical exercise and/or cycling per week OR physical job and some but <1 hour of physical exercise and/or cycling per week OR heavy manual job.^a^
Stanford Brief Activity Survey (SBAS)	2	<5 min	1. On-the-job activity (A-G responses) 2. Leisure-time activity (F-J responses)	A. If you have no job or regular work, check box A and go on to item 2. B. I spent most of the day sitting or standing. When I was at work, I did such things as writing, typing, talking on the telephone, assembling small parts, or operating a machine that takes very little exertion or strength. If I drove a car or truck while at work, I did not lift or carry anything for more than a few minutes each day. C. I spent most of the day walking or using my hands and arms in work that required moderate exertion. When I was at work, I did such things as delivering mail, patrolling on guard duty, doing mechanical work on automobiles or other large machines, house painting, or operating a machine that requires some moderate-activity work of me. If I drove a truck or lift, my job required me to lift and carry things frequently. D. I spent most of the day lifting or carrying heavy objects or moving most of my body in some other way. When I was at work, I did such things as stacking cargo or inventory, handling parts or materials, or doing work like that of a carpenter who builds structures or a gardener who does most of the work without machines. E. I spent most of the day doing hard physical labor. When I was at work, I did such things as digging or chopping with heavy tools or carrying heavy loads (bricks, for example) to the place where they were to be used. If I drove a truck or operated equipment, my job also required me to do hard physical work most of the day with only short breaks. F. Most of my leisure time was spent without very much physical activity. I mostly did things like watching television, reading, or playing cards. If I did anything else, it was likely to be light chores around the house or yard or some easygoing game like bowling or catch. Only occasionally, no more than once or twice a month, did I do anything more vigorous, like jogging, playing tennis, or active gardening. G. Weekdays, when I got home from work, I did few active things, but most weekends I was able to get outdoors for some light exercise — going for walks, playing a round of golf (without motorized carts), or doing some active chores around the house. H. Three times per week, on average, I engaged in some moderate activity, such as brisk walking or slow jogging, swimming, or riding a bike for 15–20 minutes or more, or I spent 45 minutes to an hour or more doing moderately difficulty chores, such as raking or washing windows, mowing the lawn or vacuuming, or playing games such as doubles tennis or basketball. I. During my leisure time over the past year, I engaged in a regular program of physical fitness involving some kind of heavy physical activity at least 3 times per week. Examples of heavy physical activity are jogging, running, or riding fast on a bicycle for 30 minutes or more; heavy gardening or other chores for an hour or more; active games or sports such as handball or tennis for an hour or more; or a regular program involving calisthenics and jogging or the equivalent for 30 min or more. J. Over the past year, I engaged in a regular program of physical fitness along the lines described in the last paragraph (I), but I did it almost daily — 5 or more times per week.	Inactive = A + F Light-intensity activity = A + G; B + (F or G); or C + (F or G) Moderate-intensity activity = (A, B, or C) + H; or D + (F or G) Hard-intensity activity = (A, B, or C) + I; D + (H or I); or E + F

a Questions concerning walking, housework/childcare, and gardening/do it yourself are included to allow patients to record their physical activity in these categories; however, these questions may not yield sufficiently reliable data to contribute to an understanding of overall physical activity levels.

**EVS.** EVS is a modification of the Behavioral Risk Factor Surveillance System (BRFSS) PA questions, and its development was supported by PA experts as part of the Exercise is Medicine effort in the United States (16). EVS assesses the average time spent exercising by multiplying responses on 2 self-reported questions: 1) “On average, how many days per week do you engage in moderate to strenuous exercise (like a brisk walk)?” and 2) “On average, how many minutes per day do you engage in exercise at this level?” The responses are multiplied to display minutes per day of moderate or strenuous exercise. EVS takes less than 30 seconds to administer.

**PAVS.** PAVS was created by investigators at the Department of Family and Preventive Medicine at the University of Utah, Salt Lake City, Utah, to implement in family medicine clinics in the Utah Health Research Network (19). Two questions are self-reported: 1) “How many days during the past week have you performed physical activity where your heart beats faster and your breathing is harder than normal for 30 minutes or more?” and 2) “How many days in a typical week do you perform activity such as this?” The responses are reported as days during the past week over days in a typical week, with scores ranging from 0 to 7 for each question. PAVS requires less than 30 seconds to administer and score (19).

**SNAP.** SNAP was developed as a PA assessment tool for primary care with involvement from culturally diverse health care providers, their staff, and patients (13). It was created on the basis of feedback from focus groups of providers, staff, and patients with broad cultural backgrounds from community health centers (13). Written at a fifth-grade literacy level, SNAP consists of one question: “Are you active for 30 minutes on 5 days of the week?” Respondents are given examples of activities (eg, walking, housework), and they select one answer choice: 1) “No, and I have no plans to be more active”; 2) “No, but I have been thinking about being more active”; 3) “Sometimes I am active for 30 minutes, but not all the time”; and 4) “Yes, I am active for 30 minutes on 5 days of the week.” SNAP requires less than 1 minute to complete.

**GPPAQ­.** GPPAQ is supported by the National Institute for Health and Care Excellence (NICE) (22). It includes 7 questions that cover PA involved in work; number of hours participating in various exercises, housework, childcare, gardening, and do-it-yourself activities; and walking pace. Patients are classified as inactive, moderately inactive, moderately active, or active. GPPAQ takes 1 minute to complete.

**SBAS.** SBAS was first used in a sample of mostly white, English-speaking adults aged 60 to 69 years (23) and since has been tested among middle-aged adults, Latinas, and African American women (20). It consists of 2 items that assess occupational and leisure-time PA with 5 response categories per item, representing inactive to very hard intensity PA (24). For scoring, the interaction of the occupational activity response (vertical axis, A–E) and the leisure-time PA response (horizontal axis, F–J) indicate the individual’s activity category. SBAS can be completed in less than 5 minutes.

### Use of Physical Activity Vital Sign Tools

EVS and PAVS were applied in large US health care systems to identify patients not meeting recommended PA levels. EVS was inserted into the vital sign section of the electronic medical record in Kaiser Permanente Southern California for use in primary care among adults aged 18 years or older (16,17,21). Medical assistants and nurses collected EVS responses during the outpatient visit before the provider interacted with the patient (16). PAVS was assessed in patients from 2 university-based family medicine clinics in the Utah Health Research Network (14,15,19) in clinic staff from 7 primary care clinics in the Salt Lake Valley area (13) and in electronic health records of primary care patients from Intermountain Healthcare, Salt Lake City, Utah (14). SNAP also was assessed among the sample of clinic staff. Staff were included to familiarize them with the PA assessments that they ultimately would administer to patients (13). PAVS was administered by a nurse or medical assistant at the initiation of the patient’s clinic visit (25). PAVS was examined in studies of adults included in this review but has been proposed to be appropriate for adolescents (25). GPPAQ is commonly used in the primary care setting in the United Kingdom to assess PA in adults (22). This tool is supported as part of the United Kingdom public health initiative “Let’s Get Moving” campaign and can be self-administered or administered by a health professional (22).

### Psychometric properties of Physical Activity Vital Sign Tools

**EVS.** A study of the face and discriminant validity of EVS was conducted using the 2010–2011 electronic medical records of more than 1.5 million adults in Kaiser Permanente Southern California (16). Face validity was calculated by comparing median total minutes per week of moderate to strenuous exercise from EVS with national population-based data from the 2005–2006 Nutrition Health and Examination Survey (NHANES) and 2007 California BRFSS. Using the EVS, sufficient PA (2008 PA guidelines) was lower than sufficient PA proportions of the NHANES or BRFSS (31%, 60%, and 50%, respectively), but the patterns of PA levels by age, sex, race/ethnicity, and BMI were comparable. In this same study, physical inactivity (0 min/wk) versus insufficient activity (>0 but <149 min/wk) on EVS was more likely with increasing age, among women than men, among Hispanics and non-Hispanic blacks than non-Hispanic whites, with greater disease burden (Charlson Comorbity Index of 3 vs 0), and with higher BMI.

In a study of 76 participants (38 from the United States and 38 from the United Kingdom) (17), sensitivity (59%) and specificity (77%) suggested a moderate ability of EVS to correctly identify participants as not meeting or meeting 2008 PA guidelines on the basis of 7-day accelerometry data. Accounting for sex and country, EVS compared with accelerometry data overestimated the number of minutes per week of moderate to vigorous PA on average by 66 minutes (*P* < .05).

Among 30 African American women (mean [standard deviation (SD)] age, 35.5 [5.3] y; mean [SD] BMI, 31.1 [7.8] kg/m^2^) participating in a PA intervention pilot study in Arizona (20), EVS and accelerometer-measured moderate to vigorous PA were weakly correlated (*r* = 0.27, *P* = .15 at baseline; *r* = 0.26, *P* = .17 at 8 weeks) (Table 2). Sensitivity, specificity, negative predictive value, and positive predictive value of EVS were 27%, 89%, 59%, and 68% at baseline and 33%, 74%, 38%, and 70% at 8 weeks, respectively. Participants who met versus those who did not meet the 2008 PA guidelines on EVS showed more accelerometer-measured moderate to vigorous PA (73 and 77 more minutes/week at baseline and 8 weeks, respectively).

**Table 2:** Assessment of Physical Activity Measures Review of Physical Activity Instruments as Vital Signs, 1985–2016

Physical Activity Measure	Psychometrics	Outcomes
Exercise Vital Sign (EVS)	Compared with national population-based data, the EVS in Kaiser Permanente Southern California demonstrated reasonable face and discriminant validity (N = ~1.5 million) (16). Compared with 7 days of accelerometry-measured moderate to vigorous PA, EVS has 59% sensitivity and 77% specificity (N = 76) (16,22). EVS overestimated the minutes of moderate to vigorous PA by an average of 66 minutes compared with 7 days of accelerometry data (N = 76) (16). In a sample of 30 African American women (20), Spearman correlation coefficient of EVS compared with minutes of accelerometer-measured moderate to vigorous PA: *r* = 0.27 at baseline and *r* = 0.26 at 8 week follow-up. In this same sample (20) using minutes of accelerometer-measured moderate to vigorous PA as criterion: sensitivity = 27% at baseline and 33% at follow-up; specificity = 89% at baseline and 74% at follow-up; negative predictive value = 59% at baseline and 38% at 8 weeks; positive predictive value = 68% at baseline and 70% at 8 weeks.	Compared with visits with EVS, implementation of an EVS program over 1.5 years in Kaiser Permanente Northern California was associated with greater exercise-related progress note documentation (26.2% vs 23.7%, *P* < .001) and referrals (2.1% vs 1.7%, *P* < .001) (N = 696,267) (17). Over 1.5 years, improvements were noted among practices with and without EVS for the outcomes of: frequency of physician exercise counseling (88% vs 76%, *P* < .001), relative weight loss among overweight patients (0.20 lbs, *P* < .001), and reduction in hemoglobin A1c among diabetes patients (0.1% decline, *P* < .001) (N = 696,267) (17). Among 622,897 members of the Kaiser Permanente Southern California system, greater PA on the EVS was cross-sectionally associated with favorable cardiometabolic factors. Compared with those who were inactive, women and men who were consistently active had lower diastolic blood pressure (−3.28 mm Hg and −1.79 mm Hg, respectively) and women who were active had lower systolic blood pressure (−4.60) and men who were consistently or irregularly active had lower fasting glucose, random glucose, and hemoglobin A1c compared with those who were consistently inactive (21).
Physical Activity Vital Sign (PAVS)	Greater moderate-intensity PA on PAVS was associated with lower BMI (construct validity, N = 261) (19). For concurrent validity (N = 269) (15), PAVS and Modifiable Activity Questionnaire (MAQ) results agreed almost 89.6% of the time. Good agreement for identifying those who met the 2008 PA guidelines (κ = 0.55, *P* < .001). Usual minutes per week of moderate to vigorous PA was highly correlated between PAVS and MAQ (*r* = 0.71, *P* < .001). In 34,712 electronic health records (14), patients who did not meet 2008 PA guidelines on PAVS (compared with those who met the guidelines) were more likely to have a higher body mass index (BMI, kg/m^2^)^a^ and greater disease burden defined as 50th percentile on the Charlson Comorbidity Index (OR = 1.77, 95% CI 1.56–2.00).	Odds of obesity decreased by 27% for every day of physical activity reported per typical week (*P* = .001) (N = 261) (19).
Speedy Nutrition and Physical Activity Assessment (SNAP), physical activity component	Among 45 clinic staff (13), poor agreement with accelerometry data for meeting recommendations of ≥30 min moderate to vigorous PA ≥5 d/wk (κ = 0.12, 95% CI, 0.04–0.28).	Not reported in articles identified for this review
General Practice Physical Activity Questionnaire (GPPAQ)	Compared with 7 days of accelerometer-measured moderate to vigorous PA, the GPPAQ has 46% sensitivity and 50% specificity (N = 76) (16).	Not reported in articles identified for this review
Stanford Brief Activity Survey (SBAS)	In a sample of 30 African American women (20), the Spearman correlation coefficient of SBAS compared with minutes of accelerometer-measured moderate to vigorous PA: *r* = 0.10 at baseline and *r* = 0.28 at 8 week follow-up. In this same sample (20), using minutes of accelerometer-measured moderate to vigorous PA as criterion: sensitivity was 18% at baseline and 67% at follow-up; specificity was 79% at baseline and 58% at follow-up; negative predictive value was 33% at baseline and 43% at 8 weeks; positive predictive value was 62% at baseline and 79% at 8 weeks.	Not reported in papers identified for this review

Abbreviations: CI, confidence interval; OR, odds ratio; PA, physical activity.

a Reference group, BMI 18.5–24.9 kg/m^2^; BMI 25.0–29.9 kg/m^2^, OR = 1.19 (95% CI, 1.07–1.32); BMI 30.0–34.9 kg/m^2^, OR = 1.39 (95% CI, 1.26–1.53); BMI 35.0–39.9 kg/m^2^, OR = 2.42 (95% CI, 2.09–2.81); BMI ≥40.0 kg/m^2^, OR = 3.70 (95% CI, 3.04–4.50).

**PAVS.** In a study of 261 participants in the Utah Health Research Network (19), BMI was 0.91 kg/m^2^ lower for each additional day of moderate PA reported on the PAVS in a typical week (*P* < .001), and BMI was 2.90 kg/m^2^ lower in participants reporting moderate PA 5 or more days in a typical week (compared with <5 days; *P* < .01). In a cross-sectional study of 34,712 electronic health records from Intermountain Healthcare (14), patients not meeting 2008 PA guidelines on PAVS were more likely to have a greater disease burden (50th percentile on Charlson Comorbidity Index; odds ratio [OR] = 1.77, 95% confidence interval [CI], 1.56–2.00) and a higher BMI (25.0–29.9 kg/m^2^, OR = 1.19, 95% CI, 1.07–1.32; reference group, 18.5–24.9 kg/m^2^) than patients meeting PA guidelines (Table 2).

Among 269 patients (15), the PAVS agreed almost 90% of the time with the Modifiable Activity Questionnaire (MAQ), a long questionnaire shown to be strongly associated with accelerometry measures of PA [26]), and agreement was good for identifying those who met the 2008 PA guidelines (κ = 0.55, *P* < .001). The usual minutes per week of moderate to vigorous PA was highly correlated between PAVS and MAQ (*r* = 0.71, *P* < .001).

In a study of 45 health clinic staff (13), number of days with 30 or more minutes of moderate to vigorous PA from PAVS and accelerometry data were correlated (*r* = 0.52, *P* < .001). Agreement for meeting PA guidelines was moderate between the PAVS and accelerometry data (κ = 0.46, *P* < .001).

**SNAP.** In the same study of the clinic staff (13), the number of days with 30 or more minutes of moderate to vigorous PA from accelerometry data was correlated with SNAP (*r* = 0.31, *P* < .05). Agreement for meeting the 2008 US PA guidelines between the SNAP and accelerometry data was low (κ = 0.12, *P* < .05).

**GPPAQ.** In the study of 76 adults from the United States and the United Kingdom (17), sensitivity and specificity of the GPPAQ (compared with 7 days of accelerometry data) were 46% and 50%, respectively.

**SBAS.** Among 30 African American women (20), the SBAS and accelerometer-measured moderate to vigorous PA were weakly correlated (*r* = 0.10, *P* = .59 at baseline; *r* = 0.28, *P* = .15 at 8 weeks). The sensitivity, specificity, negative predictive value, and positive predictive value of the SBAS were 18%, 79%, 33%, and 62% at baseline and 67%, 58%, 43%, and 79% at 8 weeks, respectively. Participants meeting versus not meeting the 2008 US PA guidelines on SBAS showed more accelerometer-measured PA (43 and 19 minutes more per week at baseline and 8 weeks, respectively).

### Outcomes of Physical Activity Vital Sign Tools

**EVS.** Data were collected from 696,267 Kaiser Permanente Southern California members contributing more than 1.5 million primary care visits over 1.5 years (2010–2011; mean age, 51.4 y; 52.3% women, 46.6% white, 30.1% obese, 33.9% with hypertension, 28.3% with dyslipidemia, 10.4% with diabetes, 8.9% with osteoarthritis, 7.4% with chronic obstructive pulmonary disease, and 6.0% with cardiovascular disease) (18). Practices with EVS versus those without EVS had a greater proportion of visits with exercise-related progress note documentation (26.2% vs 23.7%, adjusted OR [aOR] = 1.12, 95% CI, 1.11–1.13) and referrals (2.1% vs 1.7%, aOR = 1.14, 95% CI, 1.11–1.18). Practices with EVS versus those without EVS reported greater frequency of physician exercise counseling (88% vs 76%, *P* < .001), a relative weight loss among overweight patients (0.20 [0.12–0.28] lbs, *P* < .001), and reduction in hemoglobin A1c among patients with diabetes and baseline hemoglobin A1c higher than 7.0% (0.1% [0.07%–0.13%], *P* < .001).

In a study of 622,897 Kaiser Permanente Southern California members (21), cross-sectional associations between EVS and cardiometabolic risk factors of blood pressure, fasting glucose, random glucose, and glycosylated hemoglobin were examined (Table 2). Women and men who were consistently active versus inactive had lower diastolic blood pressure. Additionally, women who were active versus inactive had lower systolic blood pressure. Women and men who were consistently or irregularly active versus inactive had lower fasting glucose, random glucose, and hemoglobin A1c.

**PAVS.** In the study of 261 Utah Health Research Network patients (19), the odds of obesity were significantly decreased by 27% (aOR = 0.73, 95% CI, 0.60–0.89) for each day of PA in a typical week (*P* = .001). Compared with those who reported engaging in PA fewer than 5 times per week, the odds of obesity were less among participants reporting exercising 5 or more times in a typical week (aOR = 0.44, 95% CI, 0.20–0.98).

## Discussion

This review located 9 studies of PA vital sign instruments, reporting 5 tools. Although evidence to support these measures (eg, validation with accelerometry, comparisons with other measures and outcomes, testing in real-word settings) is emerging, results from this review suggest the potential effect of these tools on clinical assessment and outcomes. Generally, these tools are simple and quick to administer and could be easily incorporated into a clinic visit. The tools showed initial promise for their clinical use in identifying physical inactivity and promoting PA. Notably, the different instruments captured various aspects of PA, with some collecting broader information on overall PA and others examining work-related versus leisure-time activity. More published evidence was found for EVS and PAVS, which assess overall PA, and both were compared with 2 distinct aspects of validation: accelerometry and health outcomes. Both measures were modestly associated with accelerometry, and moderate associations are often the norm for self-report PA measures (26). Use of EVS in a health care system showed significant improvements in key health outcomes (eg, weight loss, reduced hemoglobin A1c) in less than 2 years, although these improvements were not likely clinically significant; measured changes in PA over time were not reported (18). Cross-sectional studies in health care systems showed positive associations of EVS with advantageous cardiometabolic risk factors (21) and an inverse association of PAVS with BMI (19). Only 1 of the 9 studies reviewed included participants with arthritis (18), and no studies focused on a PA tool in these patients. None of these studies had assessments of pain, physical function, activities of daily living, or quality of life as outcomes, which would be relevant to arthritis populations. The tools examined in this review appear to have questions that would be relevant to adults with arthritis, and future work should examine their utility in this group.

Few published works are available on the research of PA measures as vital sign tools, but the rate of publishing on the PA vital sign is increasing. In this expanding research area, opportunities exist to enhance our knowledge of the utility of PA vital sign measures in the clinical setting. First, research is needed to confirm the reliability and validity over time of the measures and compare the tools in different clinical populations to objective measures (eg, accelerometry data) with representative samples large enough to detect correlations and minimize bias, recognizing that adjustments may be needed to account for overestimation of PA on self-report measures. Second, longitudinal studies are needed to determine whether use of the instrument in the clinical setting results in PA change for the patient. Third, most instruments are short and appear easy to use, but our search did not find qualitative data on satisfaction with these tools in the clinical setting. Thus, comparisons of all instruments to one another is needed to determine which is the most user friendly for clinicians and patients. Fourth, these measures were examined among general adult patient groups, but further examination is necessary to determine how these measures perform among people with specific chronic diseases (ie, arthritis), those with poorer health literacy, and children and adolescents. Considering the high level of childhood obesity and its link with the escalating occurrence of chronic diseases at increasingly younger ages (25), a PA vital sign tool may help identify inadequate PA and encourage a dialogue between pediatrician, parent, and child to promote PA. Finally, other brief measures of PA that may be applicable in clinical settings with a lower health literacy level (eg, Rapid Assessment of Physical Activity [27]) were not found in our search of instruments used in clinical populations, and their utility in PA promotion could be investigated to determine whether certain instruments are more easily understood by or better capture PA data among different patient groups.

Despite the novelty of this research, inquiring about PA during clinic visits is critical for disease prevention and management, and brief vital sign tools could be considered for identifying patients who would benefit from exercise counseling. For example, assessing PA at a clinic visit for a patient with arthritis could result in referral to physical therapy, to evidence-based community programs (eg, Walk With Ease), or both. These tools should be tested with various patients to ensure they capture the essential points of PA in a manner that enhances provider–patient conversations and supports referrals to PA interventions. Given the centrality of PA for managing arthritis and its comorbid conditions, further research of this vital sign in populations that include this patient group is important.
